# Feasibility and Acceptability of a Codesigned Health Care Transition Intervention for Young People With Spinal Cord Injuries

**DOI:** 10.46292/sci22-00049

**Published:** 2023-08-16

**Authors:** Emily Alice Bray, Ajesh George, Bronwyn Everett, Yenna Salamonson, Lucie M. Ramjan

**Affiliations:** 1 School of Nursing and Midwifery, Western Sydney University, Penrith, NSW, Australia; 2 Ingham Institute Applied Medical Research, Liverpool, Australia; 3 School of Dentistry, Faculty of Medicine and Health, University of Sydney, Camperdown, Australia; 4 School of Nursing, Faculty of Science, Medicine and Health, University of Wollongong, Wollongong, Australia

**Keywords:** acceptability, codesign, feasibility, participatory action research, spinal cord injury, young people

## Abstract

**Background:**

Due in part to medical complications, adults with a pediatric onset spinal cord injury (SCI) are at higher risk of experiencing dissatisfaction with life and lower perceived physical health when compared to their peers with no disability. To support the prevention of medical complications, young people with SCI must successfully transition to adult health care. Health care transition (HCT) interventions can support young people with chronic conditions in their move to adult health care.

**Objectives:**

To evaluate the feasibility and acceptability of a web-based HCT intervention codesigned with young people with SCI and parents/caregivers.

**Methods:**

Semi-structured individual interviews were conducted online with young people with SCI and parents/caregivers who transitioned or were preparing for the transition from pediatric to adult health care. Interviews were also conducted with health care professionals. The interviews were analyzed using a hybrid deductive and inductive qualitative content analysis process. Feasibility and acceptability were measured using Bowen and colleagues’ framework, which includes eight focus areas: acceptability, demand, implementation, practicality, adaption, integration, expansion, and limited efficacy.

**Results:**

Overall, participants responded positively to the intervention and believed that it would be useful to young people with SCI and parents/caregivers. Two areas of Bowen et al.'s framework, implementation and integration, require further consideration in terms of how to embed the intervention into the current transition process.

**Conclusion:**

This study found the HCT intervention to be an innovative approach to support young people with SCI and their parent/caregivers that demonstrates promise in the areas of feasibility and acceptability.

## Introduction

Despite having educational achievements that are comparable to population norms, adults with a pediatric-onset spinal cord injury (SCI) are less likely to find employment, less likely to live independently, and more likely to report significantly lower life satisfaction and perceived physical health when compared to their peers with no disability.^1^,^2^ Although several factors influence these outcomes, medical complications have been found to be predictive of lower success.^3^ Ensuring young people with pediatric onset SCI have access to ongoing health care may help prevent medical complications and facilitate successful outcomes as adults.

Many factors can impact young people with disabilities and their successful transition to adult health care. Young and colleagues^4^ reported that for young people with cerebral palsy, spina bifida, and acquired brain injuries, transition-specific challenges include accessibility to health care services, lack of professional knowledge, limited information, and uncertainty around the transition process. For young people with SCI, resource limitations and complex navigation within adult health care services have been identified as barriers to successful health care transition (HCT).^5^ However, facilitators of a successful HCT include early provision of detailed information and extensive support as well as access to medical records, referrals to adult providers, and collaboration between adult and pediatric providers.^4^,^5^ This highlights the need for pediatric health care services to develop interventions that incorporate facilitative elements, with web-based interventions shown to be feasible and acceptable in chronic conditions.^6^-^10^

Our qualitative program of research^11^ conducted in New South Wales (NSW), Australia, built upon current literature and provided young people with SCI and parents/caregivers a platform to voice their needs and collaborate in the development of an intervention to support HCT. Our work revealed that participants were not satisfied with the transition from pediatric to adult health care services. As in Porto et al.,^5^ they reported a lack of preparation for transition and a scarcity of information about the process. Building upon these findings, young people with SCI and parents/caregivers provided clear recommendations on how to address these gaps and in response; a novel HCT intervention was codesigned and developed, the SCI Healthcare Transition website.^12^ The website provides a step-by-step guide to the HCT process, categorized by age, that includes tools (e.g., a medical summary template, transition readiness checklist, and goal-planning worksheet), tips (e.g., PDFs on questions to ask your health care team about HCT), and resources (e.g., links to services and an infographic of the HCT process) to help prepare for the move. The goal was (1) to support young people with SCI achieve greater health care independence by equipping them with knowledge of their SCI and skills to help navigate the adult health care services, (2) to support the smooth and coordinated handover from pediatric to adult health care services by providing them with information on what to expect from the HCT process, and (3) to offer links to peer support. This study evaluated the feasibility and acceptability of this codesigned HCT intervention.

## Methods

### Study context

The overarching study consisted of three phases driven by participatory action research (PAR) methodology (see **Table 1**) and aimed to codesign, develop, implement, and evaluate an HCT intervention to support young people with SCI in NSW, Australia. Briefly, Phase 1 used semi-structured interviews to explore the HCT experience of young people with SCI and parents/caregivers. Phase 2 consisted of two codesign workshops (one with young people with SCI and one with parents/caregivers), after which the SCI Healthcare Transition website was developed and then refined in two focus groups (one with young people with SCI and parents/caregivers, and one with pediatric health care professionals). The study protocol and results from the first two phases have been published elsewhere.^11^-^13^ This article presents the findings from Phase 3—the review phase.

**Table 1.** Cyclical participatory action research project phases

Phase	Steps
**Phase 1 - PLAN** *Exploring current experiences and unmet needs*	Plan Service consultations with key pediatric SCI health care services Scoping review on the feasibility of codesigned health care transition interventions
**Completed**	Act Individual semistructured interviews conducted online via Zoom
	Review Interview analysis – confirmed and refined by young people
**Phase 2 - ACT** *Co-designing the HCT intervention*	Plan Two codesign workshops
	Act Intervention development
**Completed**	Review Two refinement focus groups
**Phase 3 - REVIEW** *Implementing and evaluating the HCT intervention*	Plan Intervention refinement
	Act Individual semistructured evaluation interviews
**Reported**	Review Interview analysis

*Note:* HCT = health care transition.

### Study design

Feasibility studies support the researcher in determining whether or not an intervention should be recommended for further testing.^14^,^15^ Bowen et al.^14^ proposes eight general areas of focus; acceptability, demand, implementation, practicality, adaptation, integration, expansion, and limited efficacy.

In this qualitative feasibility study, individual semi-structured interviews explored the views and experiences of end users of the SCI Healthcare Transition website. The Care Transitions Framework,^16^ which guided development and evaluation of the intervention, and is described in further detail in Bray et al.^12^,^13^

### Recruitment and participants

Young people with SCI, aged between 14 and 25 years who had acquired a pediatric-onset SCI (at or before the age of 16), and their parents/caregivers were invited to participate. Health care professionals from key pediatric SCI support services were also recruited.

For a detailed account of recruitment for the overall study, see Bray et al.^13^ For this phase of the study, all participants who had taken part in a previous phase were first contacted via email to determine their interest to continue participating in the project. Interested participants were then contacted via phone to confirm their availability and book in the interview. Recruitment flyers were also posted on social media and sent to key pediatric SCI health care services. In the end, three young people with SCI (all female; two with tetraplegia and one with paraplegia), three parents/caregivers (one a mother of a daughter with paraplegia and two mothers of children with tetraplegia— one male and one female) were recruited. Of these six participants, four had already transitioned to adult health care, one was currently transitioning to adult health care, and one was preparing to transition to adult health care in the next few years. All but one participant had taken part in a previous phase of the larger study.

Health care professionals from key pediatric SCI support services who consulted during the PAR study were also recruited. Four health care professionals (a clinical nurse consultant, occupational therapist, pediatric rehabilitation specialist, and transition support services manager) took part in this phase of the study.

Although the sample may not be representative of the entire pediatric SCI community and those that support them, theoretical data saturation was reached following analysis of the sixth interview. Four further interviews confirmed no new information or concepts related to the study aims.

### Data collection

The interviews were conducted online by the primary researcher (E.A.B.) and were recorded and transcribed verbatim. Bowen and colleagues’^14^ eight domains of feasibility guided the development of open-ended questions (see eTable 1 for an overview of questions). These questions also addressed the Care Transitions Framework Domains – Measures of Implementation and Outcomes.

### Data analysis

A hybrid deductive and inductive qualitative content analysis process^17^ was used to create categories and subcategories. This approached allowed the eight domains of feasibility to be integral to the process of deductive coding while allowing for inductive codes to be generated from the data.

To obtain a sense of the data, E.A.B. and L.M.R. independently immersed themselves in the data, listening to the audio recordings and reading the transcripts repeatedly. Following this, in line with a deductive approach to analysis,^17^ a categorization matrix for coding based on the framework of Bowen and colleagues was created. For the inductive approach to analysis,^17^ E.A.B. and L.M.R. condensed the data into meaning units through open coding, categorization, and abstraction (see eTable 2 for an example). Together, E.A.B. and L.M.R. refined the codes and constructed preliminary subthemes. The entire research team reviewed and confirmed the final themes and subthemes.

### Ethics

Ethics approval was obtained from the Western Sydney University Human Research Ethics Committee (H14029). Participation was voluntary. Both written and verbal consent was obtained. Transcripts were de-identified, and participants were assigned a pseudonym to maintain anonymity. Young people with SCI and parents/caregivers each received a $30 e-gift card as a thank you for their time.

### Rigor and reflexivity

To maintain rigor within the study, trustworthiness of the data was enhanced through iterative questioning and paraphrasing interpretations to ascertain authenticity during the interviews.^18^ Tracking coding and category decisions and confirming these through researcher triangulation ensured credibility.^19^ Verbatim quotes supported contextualization of interpretations (credibility and authenticity).^19^ A comprehensive reflective commentary was maintained that traced the thinking processes and decisions made during the conduct of the study (dependability and confirmability).^18^ Furthermore, as an individual with an SCI, the first author engaged in personal reflexivity of assumptions.^20^

## Results

### Category 1: Acceptability – “Overall, I think it's great”

All participants described having a positive experience when using the website for the first time. Half (50%) of young people and parents/caregivers who had already transitioned to adult health care explicitly stated that they wished they had access to a website like this when they were transitioning from pediatric to adult health care services.

*I wish that I had something like this when I was transitioning from kids to adults.* Jamie, young person (YP)

#### Subcategory 1.1: Positive characteristics – “It's very easy to use, very easy to navigate”

Six out of 10 (60%) participants found the website user-friendly or easy to navigate. All young people and three out of the four (75%) health care professionals reported liking the aesthetics and interactive nature of the website. By contrast, the comments of parents/caregivers focused on the value of the content, depth of information, and credibility of the information.

*I also liked the fact that you actually shared your work with people. That adds to your credibility and…you share your story, which makes people accept your website more and trust you more because they know that you are the first source of information going through similar things and thinking from an academic perspective.* Morgan, parent/caregiver (P/C)

#### Subcategory 1.2: Areas for improvement – “more about what's missing”

Participants commented less about what areas required improvement and more about what was missing. For example, participants requested more information on peer support for parents, the National Disability Insurance Scheme,^21^ and processes within the adult health care services.

Occasionally participants mentioned items they would have liked included on the website that were already available suggesting some information needed to be clearer.

Although seven participants valued the videos and noted wanting more of them, one participant highlighted that the animated video on the homepage lacked diversity.

*To get a bit more diversity in there…different skin colours and different sex and all those sorts of things… so that a young person can see themselves somewhere in one of those cartoon characters.* Cameron, health care professional (HP)

### Category 2: Demand – “I would be absolutely telling families about this”

All young people and parents/caregivers expressed an intent to continue using the website, and all participants stated that they would recommend the website to a young person with SCI, parent/caregiver, or health care professional, confirming its acceptability.

*I'd recommend it to some of my friends, especially because some of them are transitioning into the adult service. Even, people that are going to be transitioning soonish, like 13, 14, 15-year-olds. I'd even share it to some of my healthcare professionals.* Skye, YP

### Category 3: Implementation – “It takes a bit more than just knowing it exists for people to use it”

When discussing the likelihood that the website could be implemented as planned, one health care professional believed that it was too early to tell and that the website required further testing.

*I think you have to road test the documents, the Word documents. Would young people actually use them? Because in my experience, they don't.* Cameron, HP

Additionally, one health care professional commented that it would take more than just knowing the website exists for people to use it. In addition to posters, QR codes, and advertisements on social media to spread the word about the website to young people and parents/caregivers, health care professionals need to learn about the website possibly as part of their ongoing professional development. Learning about the content of the website and how it can be used means health care professionals can then incorporate the website into their practice and conversations with young people and parents/caregivers.

### Category 4: Practicality – “I'd do it there in clinic…I'd take them through and I'd show them”

All health care professionals thought the website had good applicability to current health care processes. They believed that the website would be best employed as a reference point or ice breaker for conversations around transition to adult health care within clinics. Health care professionals could guide young people and their parents/caregivers through the website and use it to facilitate conversation. Young people and parents/caregivers confirmed that this would be ideal as they felt they would not have searched for this type of website on their own.

*If [healthcare professionals] can recommend and maybe even sit with families and go through [the website] and show them and make them aware because lots of people in these circumstances are quite lost and quite physically and emotionally drained, and they might not have patience or time or anything to research by themselves, but if someone actually goes through your website with them and point to things, that will be really useful.* Morgan, P/C

Furthermore, it was identified that the website would be helpful for those who did not want to engage in face-to-face conversation but wanted information on the HCT process.

*It also enables people who may not want to necessarily engage in that face-to-face healthcare model to visit the website and do it themselves. We acknowledge that everyone learns in different ways and has different preferences to how they would want to engage with the system and so this is just another way that they can.* Sage, HP

### Category 5: Adaption – “It can be useful for other disabilities”

All participants believed that the website could be used as a template for developing similar websites for young people with other chronic illnesses and disabilities.

*It's a beautiful template for other conditions (cerebral palsy and for spina bifida and some of our musculoskeletal conditions and our brain injury kids).* Avery, HP

### Category 6: Integration – “It fits with the current transition”

Health care professionals believed that the website fit with the current HCT process, but that integration would require pediatric SCI health care services to take ownership of the website.

*You really need the children's hospitals and [pediatric SCI services] to take that ownership because…the way I see it working best is when the use of it is modelled to [young people].* Reese, HP

Three participants also discussed the sustainability of the website and questioned what draws someone back to the website. They believed that the website needed to be living, continuously updated, and offer more information on general adolescent transitions.

*If there was information on transitioning out of home, people that have already transitioned could go back to use the website to get information. So the website would definitely appeal to more people and reach more people and target more people that have already gone through transition, but need help transitioning into different stages of their life.* Jamie, YP

### Category 7: Expansion – “There's enough generality there”

All health care professionals believed that the website could be expanded and used in other states of Australia if state specific resources were added.

*A lot of [the website] is general, but then when you get into the nitty gritty, it's New South Wales. I think for Victoria, we'd have to put in the equivalent services and so forth, which that's not hard. I mean, we could definitely do that.”* Cameron, HP

### Category 8: Limited efficacy – “Empowering”

#### Subcategory 8.1: Impact on young people – “They are not lost”

Participants believed that the website would be empowering for young people with SCI. It was thought that the website would provide young people with a better understanding of the HCT process, engage them in getting ready for adult health care services, and give them more responsibility for their health care. Moreover, it would reduce anxiety, build confidence, and assist with communication.

*It would give a lot of young people more encouragement and confidence when transitioning because they have this website to fall back on with all the information that they need about transitioning and support.* Jamie, YP

#### Subcategory 8.2: Impact on parents/caregivers – “clearer and less frightening”

Parents/caregivers stated that the website would make the HCT process clearer. This would reassure and comfort them as parents/caregivers would not only have access to transition-related information but also the knowledge that other parents/caregivers have gone through the same process.

*I think it's just more a security thing to know that there's a reference there, to make sure nothing is being overlooked and to understand that process of transitioning. It's better to have a reference like that than to be going through it with uncertainty or without knowledge about how it's going to be managed or even the types of questions that you need to ask or the services that you need to consider.* Jude, P/C

## Discussion

This study investigated the feasibility and acceptability of a web-based intervention to support young people with SCI in their transition from pediatric to adult health care services. The intervention consisted of a codesigned website, the SCI Healthcare Transition website. Results were analyzed using Bowen et al.'s framework for feasibility.^14^ Overall, the results demonstrated that the website is feasible and acceptable.

Six out of eight areas of Bowen et al.'s framework supported feasibility, including findings related to the website's acceptability, demand, practicality, adaption, expansion, and limited efficacy.

There were two areas of the framework that challenged feasibility and require further consideration: implementation and integration. Firstly, participants believed it was too early to determine if the website could be implemented as planned. It was thought that health care providers would need to be educated on the website and how it can be used in practice. Since the completion of the feasibility study, the website was launched formally to health care professionals demonstrating how it can be used to support young people in their transition to adult health care. A total of 56 individuals registered for the event, the majority of whom were health care professionals working with young people with SCI. The webinar hosted 30 attendees, and within a week of the launch the website had over 80 visitors.

Regarding the integration of the website into the current HCT process, health care professionals indicated that there would have to be some degree of buy-in from pediatric SCI health care services to ensure they continued to use and recommend the website to young people and parents/caregivers. Researchers have since addressed this concern by approaching a pediatric SCI support service in New South Wales to take ongoing responsibility and to host the link for the website.

A review that determined the scope of published literature on codesigned HCT interventions and their feasibility found 17 interventions to be feasible and acceptable,^22^ and two were codesigned websites. Coyne and colleagues^9^ assessed the feasibility of a codesigned website to support young people with long-term illnesses in their transition to adult health care. Preliminary feedback indicated that the resource was a reliable, functional, and acceptable intervention to support young people in their transition to adult health care.

Ammerlaan et al.^7^ assessed the feasibility of a codesigned website and hospital-based online portal for young people with juvenile idiopathic arthritis. Participants considered the website useful and easy to use; similar to this study, they found the videos most useful and an enjoyable way to learn. Participants indicated new information might encourage website return, and a section with news and events was added to encourage repeat visits. This sentiment was also highlighted by participants in this study; they stated that information and guidance on more of life's general adolescent transitions (i.e., moving out of home, starting university) would draw them back to the website. Interestingly, both studies highlight that despite only addressing a specific period in an adolescent's life in which they are transitioning between health care services, websites on HCT may be further strengthened by addressing more adolescent developmental milestones and providing new and updated information.

Research on the meaningful involvement of end users in disability research indicates that end users contribute positively to research through their contribution of lived experience.^23^ Furthermore, including end users in the development of eHealth applications has been shown to have a positive impact on the actual uptake of the application in daily life.^24^,^25^ Both Ammerlaan et al.^7^ and Coyne et al.^9^ highlighted that including end users in the codesign of the interventions could have attributed to its more positive outcomes. The positive outcomes of this study may also be attributed to the inclusion of end users in the collaborative development of the SCI Healthcare Transition website.

Future research should be undertaken to further evaluate the effectiveness of the website. Additional research could also take an implementation science lens^26^ to understand what influence health care professional and organizational behaviour may have to the successful implementation and integration of the website into the current HCT process.

## Limitations

A limitation of the study could be the small sample size and digital literacy of our sample. We did not achieve a representative sample. No male identifying individuals were included in the young people with SCI or parent/caregiver groups, although a young male was involved in the development phase. Additionally, we did not achieve cultural and linguistically diverse representation (including Aboriginal and Torres Strait Islander input). However, in a feasibility study, it is appropriate to have a small sample to determine whether the intervention is feasible before doing a major study.^14^

## Conclusion

The SCI Healthcare Transition website is a novel, innovative intervention designed to support young people with SCI in their transition from pediatric to adult health care services. This study has shown that the website is feasible and acceptable.
