# Supplementary material for: Feasibility and Acceptability of a Codesigned Health Care Transition Intervention for Young People With Spinal Cord Injuries
Source: Top Spinal Cord Inj Rehabil. 2023 Aug 16;29(3):89–97. doi: 10.46292/sci22-00049 (PMC10644856; doi:10.46292/sci22-00049)
Supplement: Supplementary file 1 [file i1945-5763-29-3-89_s01.pdf]

eTable 1. Feasibility questions for young people/parents/caregivers

Area of focus	The feasibility study asks …	Sample outcomes of interest	Guiding questions
Acceptability	The extent to which the HCT intervention is judged as suitable, satisfying, or attractive by young people with SCI and their parents/ caregivers.	• Satisfaction<br>• Intent to continue use<br>• Perceived appropriateness	• How would you describe your experience using the website?<br>• What aspects did you like the most?<br>• What aspects did you least like?<br>(Prompts: navigation, information, layout, videos, graphics, tasks, accuracy, reliability)
Demand	To what extent is the HCT intervention likely to be used?	• Expressed interest or intention to use<br>• Perceived demand	• Would you continue to use the website?<br>• Would you recommend this website to someone else?<br>(Prompts: What would motivate you to continue using the website?)
Implementation	The extent, likelihood, and manner in which the HCT intervention can be fully implemented as proposed.	• Amount, type of resources needed to implement	• N/A
Practicality	The extent to which the HCT intervention can be delivered by SCI health care service providers.	• Factors affecting implementation ease or difficulty	• How could health care providers support the delivery and use of this intervention?<br>• Can you tell me if you experienced any difficulties using the website?<br>• What factors made it easy for you to use the website?<br>(Prompts: How easy was it to locate the information and tasks? How intuitive was it to navigate through it? Were you able to go back and forth through the information and tasks?)
Adaption	Could you accommodate the HCT intervention context and requirements in a different format, media, or population?	• Perceived degree to which similar outcomes are obtained in new format or for a different population	• On what platforms were you able to access this intervention? (Prompts: mobile, iPad, tablet, laptop/ computer, other)<br>• Are there other populations you think this website could work or be adapted for use? Please explain

(continues)

This supplementary digital material accompanies:
Top Spinal Cord Inj Rehabil 2023;29(3):89-97
© 2023 American Spinal Injury Association
www.asia-spinalinjury.org
doi: 10.46292/sci22-00049

eTable 1. Feasibility questions for young people/parents/caregivers *(cont.)*

**Area of focus**	**The feasibility study asks …**	**Sample outcomes of interest**	**Guiding questions**
Integration	Would SCI health care service providers be able to integrate the HCT intervention into the existing transition process?	• Perceived fit with infrastructure<br>• Perceived sustainability	• How do you envisage the intervention fitting in with the current transition process?<br>• How could health care providers promote the intervention during transition? And into the future for its sustainability?
Expansion	Potential success of implementing the HCT intervention in a different setting (e.g., state).	• Perceived fit with organizational goals and culture	• N/A
Limited efficacy	Testing of the HCT intervention in a limited way.	• Intended effects of program or process on key intermediate variables	• What do you see the impact of this intervention being for YP with SCI in the short term? Medium term?<br>• What do you see the impact of this intervention being for parents/ caregivers in the short term? Medium term?

Note: HCT = health care transition; YP = young people.

eTable 2. Examples of meaning units, condensed meaning units, codes, subcategories, and categories

Meaning unit	Condensed meaning unit	Code	Subcategory	Category
"The other thing is whether it's possible I know it's already been built but whether it's possible, because we're doing something similar in another area, to get a bit more diversity in there. You know, I think so different skin colors and different sex and all those sorts of things is good if it's possible. Just diversity is always good so that a young person can, sort of, see themselves, I guess, somewhere in one of those cartoon characters."	*"To get a bit more diversity in there…different skin colors and different sex and all those sorts of things… so that a young person can see themselves somewhere in one of those cartoon characters"*	How the website could be improved	Areas for improvement – "more about what's missing"	Acceptability – "Overall, I think it's great"

Note: eTable 2 provides an example of how the data were condensed into meaning units, coded, and categorized.
